# Serious Adverse Events and Laboratory Monitoring Regimens for Outpatient Parenteral Antimicrobial Therapy With Daptomycin

**DOI:** 10.1093/ofid/ofag254

**Published:** 2026-06-08

**Authors:** Shawnalyn W Sunagawa, Richard Hankins, Jordan Kuck, Sandra Frimpong, Nicolas Cortes-Penfield, Elizabeth Lyden, Melissa LeMaster, Kathleen Nguyen, Bryan T Alexander, Molly M Miller

**Affiliations:** Department of Pharmacy Practice and Science, College of Pharmacy, University of Nebraska Medical Center, Omaha, Nebraska, USA; Department of Pharmaceutical and Nutrition Care, Nebraska Medicine, Omaha, Nebraska, USA; Division of Infectious Diseases, College of Medicine, University of Nebraska Medical Center, Omaha, Nebraska, USA; Department of Pharmaceutical and Nutrition Care, Nebraska Medicine, Omaha, Nebraska, USA; Department of Internal Medicine, College of Medicine, University of Nebraska Medical Center, Omaha, Nebraska, USA; Division of Infectious Diseases, College of Medicine, University of Nebraska Medical Center, Omaha, Nebraska, USA; Department of Biostatistics, College of Public Health, University of Nebraska Medical Center, Omaha, Nebraska, USA; Department of Pharmaceutical and Nutrition Care, Nebraska Medicine, Omaha, Nebraska, USA; Department of Pharmacy Practice and Science, College of Pharmacy, University of Nebraska Medical Center, Omaha, Nebraska, USA; Department of Pharmaceutical and Nutrition Care, Nebraska Medicine, Omaha, Nebraska, USA; Department of Pharmaceutical and Nutrition Care, Nebraska Medicine, Omaha, Nebraska, USA

**Keywords:** adverse events, daptomycin, laboratory techniques and procedures, outpatient parenteral antimicrobial therapy

## Abstract

The optimal laboratory monitoring frequency for outpatient parenteral antimicrobial therapy-related adverse events (OPAT-AEs) during daptomycin therapy is not well defined. We identified just 6.6 OPAT-AEs per 1000 sets of weekly laboratory tests in this population, suggesting that less intensive laboratory monitoring may be reasonable in patients receiving daptomycin for OPAT.

Outpatient parenteral antimicrobial therapy (OPAT) benefits patients and hospital systems by decreasing hospital lengths of stay, healthcare-associated costs, and improving patient satisfaction [1–3]. The Nebraska Medicine OPAT program was established in 2019 and consists of a multidisciplinary team of infectious diseases clinicians, who via electronic consultation, evaluate and monitor OPAT regimens. Current Infectious Diseases Society of America (IDSA) guidelines recommend frequent, often weekly, laboratory monitoring during OPAT [1]. However, these guidelines reference no studies directly assessing which or how often specific labs should be ordered for monitoring individual antimicrobials. We previously assessed laboratory monitoring and OPAT-related adverse events (OPAT-AEs) with cefazolin and ceftriaxone, observing just 2.7 OPAT-AEs per 1000 sets of weekly laboratory tests, leading us to reduce the laboratory monitoring frequency for these agents [4].

Opportunities exist to further align current clinical guidelines for OPAT laboratory monitoring to the OPAT-AE risks of individual antimicrobial agents. Some studies have demonstrated higher daptomycin OPAT-AE rates of ∼2–35%, but definitions of what constituted a clinically significant AE differed, and these studies failed to report whether OPAT-AEs were detected based on laboratory monitoring [3, 5–8]. A previous OPAT-AE propensity score-matched cohort study demonstrated that antimicrobial-associated AEs for daptomycin versus vancomycin were 3.2 versus 7.7 per 1000 OPAT days, respectively (*P* = .02), yet these agents have the same recommended weekly laboratory monitoring frequency [6]. Daptomycin's improved safety profile, compared with vancomycin, and convenient once daily dosing, has made it a preferred treatment agent for gram-positive infections in OPAT. However, data-driven laboratory monitoring frequency recommendations are lacking. Thus, the aim of this study was to assess OPAT-AE rates for patients receiving daptomycin via OPAT to better define the appropriate frequency for OPAT laboratory monitoring.

## METHODS

We retrospectively evaluated patients followed by our OPAT program who received outpatient daptomycin between 1 June 2020 and 30 June 2024. Patients receiving multiple intravenous antimicrobials were excluded; however, patients receiving concomitant oral antibiotics were included. The primary outcome was incidence of clinically significant OPAT-AEs, defined as drug-associated AEs (eg, abnormal labs, allergic reactions, or *Clostridioides difficile* infection) leading to a change in antimicrobial therapy or any catheter-associated AE requiring intervention. Secondary outcomes included time from start of antimicrobial therapy to clinically significant OPAT-AE, unplanned healthcare utilization (eg, emergency department visits, readmission), and OPAT actions performed to obtain or clarify laboratory monitoring data. Our institutional review board deemed this a quality improvement project exempt from review.

Each OPAT-AE was independently adjudicated by 2 investigators. We performed descriptive statistics as well as Fisher exact and independent sample t-tests to assess univariate associations. Multivariable logistic regression with backward selection was used to determine if variables significant on univariate analysis remained independently predictive of OPAT-AEs after adjusting for other covariates in the model. All analysis was performed using SAS, version 9.4 (SAS Institute Inc., Cary, NC), and *P* values <0.05 were considered statistically significant.

## RESULTS

Our cohort included 330 courses of daptomycin comprising 11 820 days of therapy and a total 1526 sets of weekly labs, typically including a complete blood count with differential, serum chemistries with or without liver function tests, and a creatinine kinase (CK), of which 97.2% were reviewed within 72 hours from lab draw. Of the 330 courses, 219 (66%) required OPAT team actions to obtain missing labs for review on at least 1 occasion. The median (interquartile range [IQR]) dose of daptomycin was 6 mg/kg (5.9, 6.5) and the median total duration of therapy was 38 days (27, 42). Most patients completed OPAT via home health care (64%), and the most common indication for OPAT was osteomyelitis (27%). Additional cohort characteristics are listed in Table 1 and Supplementary Table 1.

**Table 1.** OPAT Cohort Characteristics

Cohort Characteristics	N = 330^a^
Male, n (%)	198 (60%)
Age (years), median (IQR)	61 (47, 69)
Weight (kg), median (IQR)	88 (72, 107)
BMI (kg/m^2^), median (IQR)	29.7 (24.6, 35.5)
Charlson Comorbidity Index Score, median (IQR)	1 (0,3)
Potential interacting medications, n (%)	
Statin therapy	115 (35)
Antihistamine	71 (22)
Initial daptomycin dose (mg), median (IQR)	550 (475, 675)
Actual body weight dose (mg/kg), median (IQR)	6 (5.9, 6.5)
Total duration of therapy (days), median (IQR)	38 (27, 42)
Duration of exposure to daptomycin (days), median (IQR)	32 (18, 41)
Vascular access, n (%)	
PICC/CVC	303 (91.8%)
Midline	25 (7.6%)
Hemodialysis line	1 (0.3%)
Peripheral	1 (0.3%)
OPAT modality, n (%)	
Home health care	211 (63.9%)
Infusion center	65 (19.7%)
Skilled nursing facility/long term acute care facility	50 (15.2%)
Hemodialysis	2 (0.6%)
Other	2 (0.6%)
Sources of infection, n (%)	
Osteomyelitis	90 (27.3%)
Prosthetic joint infection/septic arthritis	76 (23%)
Primary/catheter related bloodstream infection	60 (18.2%)
Infective endocarditis/vascular/cardiac device infection	43 (13.0%)
Skin and soft tissue/trauma/wound infection	28 (8.5%)
Intra-abdominal infection	9 (2.7%)
Epidural abscess	7 (2.1%)
Pneumonia/lung abscess/empyema	7 (2.1%)
Central nervous system/neurological infection	3 (0.9%)
Other^b^	7 (2.1%)

Abbreviations: BMI, body mass index; CVC, central venous catheter; IQR, interquartile range; kg, kilogram; kg/m^2^, kilograms per meters squared; mg, milligrams; mg/kg, milligrams per kilogram; OPAT, outpatient parenteral antimicrobial therapy; PICC, peripherally inserted central catheter.

^a^318 Unique patients, 12 patients had >1 course of daptomycin during the study period.

^b^Urological, peritonitis/pericarditis, mastoiditis, sinusitis, spinal cord stimulator infection.

We identified 17 (5.2%) drug-associated OPAT AEs, with only 10 (3%) recognized via routine laboratory monitoring after a median (IQR) of 16 days (14, 20), and only 2 resulting in unplanned healthcare utilization. Descriptions of the abnormal laboratory-associated OPAT-AEs are listed in Table 2. The overall incidence of laboratory monitoring-associated clinically significant OPAT-AEs was 6.6 per 1000 sets of weekly labs. Clinically significant catheter-associated OPAT-AEs complicated 14 courses (4%), developing after a median (IQR) of 17 days (11, 27). Additional secondary outcomes are listed in Table 2. Univariate and multivariate logistic regressions are listed in Supplementary Tables 2 and 3; we noted no statistically significant differences in duration of therapy, daptomycin dose (mg or mg/kg), weight, or concomitant statin therapy between the groups with or without clinically significant drug-associated OPAT-AEs. In the univariate analysis, mean estimated glomerular filtration rate was significantly lower in those with drug-associated OPAT-AEs (64.4. vs 75.8; *P* = .003); however, this was not statistically significant in the multivariate analysis (Odds Ratio [OR] (95% Confidence Interval [CI]) = 0.98 (0.958, 1.003); *P* = .086).

**Table 2.** Primary and Secondary Outcomes

Primary/Secondary Outcomes	N = 330^a^
Total adverse event, n (%)	31 (9.4%)
Drug associated adverse events, n (%)	17 (5.2%)
Abnormal labs, n (%)	
Creatinine kinase	9 (2.7%)
Eosinophils	1 (0.3%)
Allergic reactions, n (%)	7 (2.1%)
Catheter associated adverse event, n (%)	14 (4.2%)
Time from start of therapy to adverse events days, median (IQR)	17 (12, 23)
Drug associated adverse events	17 (14, 21)
Drug associated abnormal lab adverse events	16 (14, 20)
Catheter associated adverse events	17 (11, 27)
Time between therapy start and peak CK (days), median (IQR)	19 (10, 28)
Time between therapy start and peak eosinophils (days), median (IQR)	14 (6, 24)
All-cause emergency department visit on treatment, n (%)	54 (16%)
All-cause readmission on treatment, n (%)	62 (19%)
All-cause 30-d hospital readmission, n (%)	59 (18%)
Infection related 30-d hospital readmission, n (%)	24 (7%)
OPAT documentation^b^	219 (66%)

Abbreviations: CK, Creatine Kinase; IQR, interquartile range; n, number.

^a^318 Unique patients, 12 patients had >1 course of daptomycin during the study period.

^b^OPAT documentation represents the total number of interventions in this cohort. Note that some courses of therapy required multiple interventions.

## DISCUSSION

To our knowledge, this is the first study specifically assessing the incidence of clinically significant laboratory-associated OPAT-AEs for daptomycin. Across over 300 courses of therapy given for a median of over 35 days and dose of 6 mg/kg, our abnormal laboratory-associated OPAT-AE rate was only 3%. Furthermore, approximately half of the drug-associated OPAT-AEs were not identifiable via routine laboratory monitoring. Together, this suggests that weekly laboratory monitoring for daptomycin via OPAT is excessive, with 153 sets of weekly labs needed to detect 1 OPAT-AE leading to a change in antimicrobial therapy.

Strengths of this study include a standardized definition for clinically significant drug-associated OPAT-AEs, which is consistent with our prior work [4]. Additionally, nearly all weekly labs (97.2%) were appropriately obtained and reviewed within 72 hours of lab draw, making it unlikely that a clinically significant abnormal lab-associated OPAT-AE was missed. Thus, the 3% incidence of OPAT-AEs detected via laboratory monitoring likely reflects a realistic incidence of these daptomycin OPAT-AEs. Notably, even this OPAT-AE rate may be inflated, as several of these changes in therapy were made for modest CK elevations below the package insert recommendations for asymptomatic discontinuation and may not have been strictly necessary (Supplementary Table 4). However, we included them as OPAT-AEs, to adhere to our definition and provide a conservative estimate of the value of daptomycin safety lab monitoring. We also note that 219 (66%) of these courses of therapy required intervention by our OPAT team to obtain initially missing labs and/or clarify, adjust, or order labs to ensure correct guideline-recommended monitoring. This represents a high burden of OPAT team resource utilization (not to mention the nursing time spent in obtaining these labs and the impact of obtaining frequent labs on patients) producing little clinical benefit.

We recognize that other studies suggest certain laboratory monitoring, including CK and eosinophils, might allow for earlier identification of severe OPAT-AEs like rhabdomyolysis and eosinophilic pneumonia [5, 9–12]. However, these are uncommon outcomes of daptomycin therapy [5, 7, 13, 14]. Moreover, in our cohort, only 1 patient with an elevated CK leading to discontinuation of daptomycin was symptomatic, the other patients were asymptomatic, but 4 patients had CK >2000 units/L (the FDA daptomycin package insert recommendation for when therapy should be discontinued based on elevated CK without symptoms, Supplementary Table 4), suggesting that routine CK monitoring may have more often driven potentially inappropriate treatment modifications [15]. If assessing discontinuation of daptomycin based on the FDA package insert recommendations, this represents just 3.3 clinically significant OPAT-AEs per 1000 sets of weekly labs, which is aligned with our previous cefazolin and ceftriaxone OPAT-AE data [4]. These data suggest that well-defined institutional approaches to daptomycin OPAT-AE management (ie, CK cutoffs for increased monitoring, holding doses, and discontinuing therapy) may aid in standardizing OPAT-AE reporting. Similarly, of our 7 patients who had therapy discontinued due to suspected eosinophilic pneumonia (classified as allergic reactions in Table 2), 6 did not have elevated peripheral eosinophilia detected by routine outpatient laboratory monitoring and the only 1 with elevated eosinophils detected by laboratory monitoring did not have any symptoms or imaging consistent with eosinophilic pneumonia but had daptomycin discontinued as a precautionary measure. It is unclear whether this patient would have developed clinical eosinophilic pneumonia. For these drug-associated OPAT-AEs, all cases occurred ≥14 days after the start of therapy, strengthening the case that routine laboratory monitoring may not be necessary for shorter durations.

Limitations of our study include its retrospective, single-institution design, and our presumption of causation in deeming clinically significant OPAT-AEs related to the patient's daptomycin via OPAT. However, each OPAT-AE was independently adjudicated by investigators as reasonably having causation with daptomycin (Supplementary Table 5). Due to the low number of laboratory-associated OPAT-AEs, multivariate modeling was not powered to capture true associations for this subgroup, so this exploratory analysis was only performed for all clinically significant drug-associated OPAT-AEs. Additionally, while not statistically significant, there was a signal toward higher initial daptomycin doses (mg) being associated with greater likelihood of OPAT-AE (OR [95% CI] = 1.006 [1, 1.012]; *P* = .052), which is aligned with previous literature demonstrating higher AE rates with increasing doses of daptomycin [3, 5–8]. We were also unable to stratify our concomitant statin data in this population due to the lower number of drug-associated OPAT-AEs, so we are unable to comment on if certain statins combined with daptomycin are more likely to cause elevated CKs, as described in previous literature [10–12]. However, for additional transparency and research, these data are provided in Supplementary Table 6.

In conclusion, in our cohort, a median daptomycin dose of 6 mg/kg was well-tolerated during OPAT, with few OPAT-AEs occurring mainly beyond 2 weeks of therapy. We identified just 6.6 clinically significant OPAT-AEs per 1000 sets of weekly labs, suggesting that routine weekly laboratory monitoring for daptomycin in the OPAT setting is excessive. Less intensive laboratory monitoring strategies than recommended by current IDSA guidelines are likely safe and should be further explored.

## Supplementary Material

ofag254_Supplementary_Data

## Supplementary Data

Supplementary materials are available at *Open Forum Infectious Diseases* online. Consisting of data provided by the authors to benefit the reader, the posted materials are not copyedited and are the sole responsibility of the authors, so questions or comments should be addressed to the corresponding author.

## References

[1] Norris AH, Shrestha NK, Allison GM, et al Infectious Diseases Society of America clinical practice guideline for the management of outpatient parenteral antimicrobial therapy. Clin Infect Dis 2019; 68:E1–35. 10.1093/cid/ciy745 30423035

[2] Hale CM, Steele JM, Seabury RW, Miller CD. Characterization of drug-related problems occurring in patients receiving outpatient antimicrobial therapy. J Pharm Pract 2017; 30:600–5. 28110617 10.1177/0897190016688771

[3] Keller SC, Williams D, Gavgani M, et al Rates of and risk factors for adverse drug events in outpatient parenteral antimicrobial therapy. Clin Infect Dis 2018; 66:11–9. 29020202 10.1093/cid/cix733 PMC5848264

[4] Sunagawa SW, Arduser S, Miller MM, et al Serious adverse events and laboratory monitoring regimens for outpatient parenteral antimicrobial therapy with cefazolin and ceftriaxone. Open Forum Infect Dis 2023; 10:ofad606. 10.1093/ofid/ofad606 PMC10727193 38111751

[5] Seaton RA, Gonzalez-Ramallo VJ, Prisco V, et al Daptomycin for outpatient parenteral antibiotic therapy: a European registry experience. Int J Antimicrob Agents 2013; 41:468–72. 23473943 10.1016/j.ijantimicag.2013.01.019

[6] Shrestha NK, Mason P, Gordon SM, et al Adverse events, healthcare interventions and healthcare utilization during home infusion therapy with daptomycin and vancomycin: a propensity score-matched cohort study. J Antimicrob Chemother 2014; 69:1407–15. 24398341 10.1093/jac/dkt512

[7] Lanier K, Jawanda J, Cid A. Adverse events leading to discontinuation of daptomycin versus vancomycin in outpatient parenteral antimicrobial therapy: a retrospective cohort study. JAC Antimicrob Resist 2025; 7:dlaf227. 10.1093/jacamr/dlaf227 PMC12661527 41321659

[8] Wiley MB, Bennett KK, Siegrist EA, Neely SB, Sassine J, White BP. Incidence and risk factors for musculoskeletal adverse effects associated with daptomycin in patients receiving outpatient parenteral antimicrobial therapy. Antimicrob Steward Healthc Epidemiol 2025; 5:e169. 40765630 10.1017/ash.2025.10087 PMC12322776

[9] West KA, Sheeti A, MacKay T, Forrest K, N G. Eosinophilic syndromes associated with daptomycin use: re-exposure hypersensitivity pneumonitis and prior peripheral eosinophilia. Open Forum Infect Dis 2022; 9:ofac065. 10.1093/ofid/ofac065 PMC8926001 35308486

[10] Imai S, Kashiwagi H, Sato Y, Miyai T, Sugawara M, Takekuma Y. Factors affecting creatine phosphokinase elevation during daptomycin therapy using a combination of machine learning and conventional methods. Br J Clin Pharmacol 2022; 88:1211–22. 34436795 10.1111/bcp.15063

[11] Shiraishi C, Kato H, Ogura T, Iwamoto T. Association between age and onset of daptomycin-induced adverse events using the U.S. Food and drug administration adverse event reporting system. J Infect Chemother 2025; 31:102501. 39209260 10.1016/j.jiac.2024.08.016

[12] Cid AN, Gibson HD, Arnoczy GS, et al Incidence of rhabdomyolysis in patients receiving daptomycin in a community hospital OPAT program. Open Forum Infect Dis 2025; 12(Supp 1):ofae631.2029.

[13] Food and Drug Adminsitration . FDA adverse events reporting system (FAERS) public dashboard for drugs and biologics. 2025. Available at: https://fis.fda.gov/sense/app/95239e26-e0be-42d9-a960-9a5f7f1c25ee/sheet/7a47a261-d58b-4203-a8aa-6d3021737452/state/analysis. Accessed 14 December 2025.

[14] Gidari A, Pallotto C, Francisci D. Daptomycin eosinophilic pneumonia, a systematic review of the literature and case series. Infection 2024; 52:2145–68. 39028390 10.1007/s15010-024-02349-z PMC11621199

[15] Food and Drug Administration . *Daptomycin*. 2020. https://www.accessdata.fda.gov/drugsatfda_docs/label/2020/208385s005lbl.pdf. Accessed 14 December 2025.

