# Supplementary material for: Serious Adverse Events and Laboratory Monitoring Regimens for Outpatient Parenteral Antimicrobial Therapy With Daptomycin
Source: Open Forum Infect Dis. 2026 Jun 8;13(6):ofag254. doi: 10.1093/ofid/ofag254 (PMC13243913; doi:10.1093/ofid/ofag254)
Supplement: ofag254_Supplementary_Data [file ofag254_supplementary_data.docx]

**Serious Adverse Events and Laboratory Monitoring Regimens for Outpatient Parenteral Antimicrobial Therapy with Daptomycin** – Supplemental Materials

**Supplemental Material**

**Supplemental Table 1:** Pathogens Isolated

| **Pathogen** | **N = 330** |
| --- | --- |
| Methicillin-resistant *Staphylococcus aureus* | 79 (23.9%) |
| Coagulase-negative *Staphylococcus* spp. | 71 (21.5%) |
| Methicillin-susceptible *Staphylococcus aureus* | 68 (20.6%) |
| Polymicrobial | 58 (17.6%) |
| *Enterococcus* spp. | 26 (7.9%) |
| Culture negative | 15 (4.5%) |
| *Streptococcus* spp. | 3 (0.9%) |
| *Cutibacterium acnes* | 2 (0.6%) |
| Other | 8 (2.4%) |

**Supplemental Table 2:** Univariate Analysis

| **Risk Factors for Drug Related Adverse Events** | | | |
| --- | --- | --- | --- |
|  | **No Drug-Associated AE**  **(n = 313)** | **Drug-Associated AE**  **(n = 17)** | **p-value** |
| Weight (kg), mean + SD | 91 + 28.2 | 106.8 + 31.6 | 0.060 |
| BMI (kg/m^2^), mean + SD | 30.7 + 9.2 | 35.6 + 9.7 | 0.059 |
| Initial daptomycin dose (mg), mean + SD | 582 + 178 | 670 + 187 | 0.076 |
| Actual body weight dose (mg/kg) | 6.5 + 1.4 | 6.2 + 0.7 | 0.302 |
| GFR on Discharge, mean + SD | 75.8 + 29.4 | 64.4 + 12.8 | 0.003 |
| Statin therapy, n (%) | 100 (33.4%) | 9 (52.9%) | 0.118 |
| Duration of therapy (days), mean + SD | 35.4 + 19.1 | 40.4 + 16.6 | 0.253 |

**Abbreviation:** AE: adverse event, BMI: body mass index, CI: confidence interval, kg: kilograms, m: meters, mg: milligrams, GFR: glomerular filtration rate, SD: standard deviation

**Supplemental Table 3:** Multivariate Analysis

| **Risk Factors for Drug Related Adverse Events** | | |
| --- | --- | --- |
| **Multivariate Analysis** | | |
|  | Odds Ratio (95% CI) | p-value |
| BMI (kg/m^2^) | 0.978 (0.879, 1.089) | 0.684 |
| Initial daptomycin dose (mg) | 1.006 (1, 1.012) | 0.052 |
| Actual body weight dose (mg/kg) | 0.655 (0.308, 1.393) | 0.272 |
| GFR on discharge | 0.98 (0.958, 1.003) | 0.086 |
| Statin therapy | 2.327 (0.78, 6.943) | 0.130 |

**Abbreviation:** BMI: body mass index, CI: confidence interval, kg: kilograms, m: meters; mg: milligrams, GFR: glomerular filtration rate, SD: standard deviation

**Supplemental Table 4:** Drug-Associated Abnormal Adverse Events, Elevated Creatinine Kinase per FDA Package Label^a^ and Eosinophilic Pneumonia requiring Treatment

| **Drug-Associated Adverse Events** | **N = 330** |
| --- | --- |
| Elevated creatinine kinase^b^ | 5 (1.5%) |
| Eosinophilic pneumonia^c^ | 4 (1.2%) |

^a^ Elevated creatinine kinase > 1000 units/L with signs/symptoms associated with rhabdomyolysis or creatinine kinase > 2000 units/L and asymptomatic

^b^ None of the patients with elevated creatinine kinases had daptomycin doses > 6 mg/kg. The 2 patients with daptomycin doses > 6 mg/kg did not meet FDA package label recommendations for discontinuation of daptomycin (patient 7.1 mg/kg dose had a creatinine kinase elevation from 10 units/L to 622 units/L with no signs/symptoms after 14 days of therapy; patient with 8.2 mg/kg dose had a creatinine kinase elevation from 36 units/L to 70 units/L with no signs/symptoms after 35 days of therapy).

^c^ Only 1 patient had elevated peripheral eosinophils detected by laboratory monitoring, the other 3 patients were diagnosed by clinical symptoms and radiographic evidence (labeled as “allergic reactions” in Table 2). All 4 patients were treated with steroids without any additional complications.

**Supplemental Table 5:** Naranjo Adverse Drug Reaction Probability Scale Scores for Drug-Associated OPAT-AEs

| **OPAT-AE Number** | **Naranjo Score** | **Naranjo Score Interpretation** |
| --- | --- | --- |
| 1 | 3 | Possible |
| 2 | 5 | Probable |
| 3 | 5 | Probable |
| 4 | 4 | Possible |
| 5 | 5 | Probable |
| 6 | 5 | Probable |
| 7 | 5 | Probable |
| 8 | 4 | Probable |
| 9 | 5 | Probable |
| 10 | 5 | Probable |
| 11 | 4 | Possible |
| 12 | 5 | Probable |
| 13 | 5 | Probable |
| 14 | 6 | Probable |
| 15 | 4 | Possible |
| 16 | 4 | Possible |
| 17 | 5 | Probable |

**Supplemental Table 6:** Concomitant Statin Therapy in Patients with Laboratory-Associated Adverse Events

| **Statin** | **N = 9** |
| --- | --- |
| Pravastatin 40 mg daily | 2 (22%) |
| Simvastatin 40 mg daily | 1 (11%) |
| Atorvastatin 40 mg daily | 1 (11%) |
| None | 5 (56%) |
